# Postmortem fetal magnetic resonance imaging: where do we stand?

**DOI:** 10.1007/s13244-018-0627-0

**Published:** 2018-06-04

**Authors:** Aurélie D’Hondt, Marie Cassart, Raymond De Maubeuge, Gustavo Soto Ares, Jacques Rommens, E. Fred Avni

**Affiliations:** 1 Department of Radiology, Hôpital Delta (CHIREC), Boulevard du Triomphe 201, 1160 Auderghem, Belgium; 2 Department of Radiology, Hôpital Ixelles, Rue Jean Paquot 63, 1050 Ixelles, Belgium; 3 Department of Neuroradiology, Hôpital Roger Salengro, Avenue du Professeur Emile Laine, 59037 Lille, France

**Keywords:** Fetus, Postmortem, Magnetic resonance imaging, Autopsy, Perinatal

## Abstract

**Abstract:**

Postmortem fetal magnetic resonance imaging (PMFMRI) is increasingly used thanks to its good overall concordance with histology paralleling the rising incidence of parental refusal of autopsy. The technique could become a routine clinical examination but it needs to be standardized and conducted by trained radiologists. Such radiologists should be aware of not only the (congenital and acquired) anomalies that can involve the fetus, but also of the “physiological” postmortem changes. In this article, we intend to focus on the contribution of PMFMRI based on the existing literature and on our own experience, as we presently perform the technique routinely in our clinical practice.

**Key Points:**

• *Concordance rates between PMFMRI and autopsy are high for detecting fetal pathologies.*

• *PMFMRI is more acceptable for parents than traditional autopsy.*

• *PMFMRI is becoming widely used as a part of the postmortem investigations.*

• *A dedicated radiologist needs to learn to interpret correctly a PMFMRI.*

• *PMFMRI can be easily realized in daily clinical practice.*

## General considerations

Although contemporary prenatal testing has improved the recognition of fetal anomalies, autopsy remains a valuable tool by providing diagnosis or clarification of some prenatal findings in 16% of cases [1]. Furthermore, it has been shown that autopsy provides important information decisive for genetic counseling in over 50% of cases [2].

In the past several decades, the number of terminations of pregnancies has increased secondary to the development of prenatal diagnosis [3]. During the same period, fetal and neonatal autopsy rates have decreased worldwide [4]. This resulted in a loss of major information that could have been used to counsel clinicians and parents regarding future pregnancies [5]. This reduction is mainly due to parental refusal of the autopsies; the reasons for their objection are (among others): religious considerations, fear of disfiguration of the dead fetus, and delay in funeral plans [6, 7]. This has brought about the development of less invasive techniques for the analysis of dead fetuses. Postmortem fetal magnetic resonance imaging (PMFMRI) has been shown to be significantly more acceptable for parents and many healthcare professionals [6]; therefore, the demand for such (less invasive) imaging examinations has increased.

Besides PMFMRI, other postmortem imaging modalities have been increasingly used or been developed, including conventional radiographs, ultrasonography (US), and computed tomography (CT). Each of these techniques has its own advantages and limitations. Still, to date, no or little established standardized guidelines have been defined for perinatal and pediatric postmortem imaging. Across European countries, there is no unique approach to determine which subpopulation of postmortem fetuses should be imaged and with what modality [8]. This will need to be defined in the near future.

PMFMRI has an overall high negative predictive value and can be used as a first intention/screening tool. A discussion between experienced perinatal radiologists, fetal pathologists, and geneticists could then select which cases would require full or selective autopsy [5]. This will be particularly important for central nervous system (CNS) anomalies [9].

For some authors, gestational age and body weight influence the diagnostic accuracy of PMFMRI. Jawad et al. [10] used the cut-off of 500 g. In their series, they demonstrated that PMFMRI provides diagnostic images in 90% of fetuses with a body weight > 535 g, opposed to less than 50% of fetuses with a body weight < 122 g. Therefore, they concluded that 500 g should be the limit for PMFMRI at 1.5 T. Their experience is not completely confirmed by ours. We do perform second trimester PMFMRI and we almost always have diagnostic images for fetuses with low body weight (our smallest fetus weighed 140 g at 15 weeks gestation) [11]. Furthermore, the prenatal diagnosis of malformations becomes more accurate in small fetuses and termination of second trimester pregnancies tends to increase. It will, therefore, be important to further develop sequences adapted to small fetuses.

In order to optimize the contribution of PMFMRI, additional information from non-invasive examinations are required, including detailed external examination, skeletal radiographs, placental analysis, and genetic testing [9].

The aims of the present overview are to summarize the present (and hypothesize the future) utility of PMFMRI based on the presently available literature and on our own experience, as we perform it routinely in daily clinical practice.

## Indications of PMFMRI

Based on the literature gathered to date and on our own experience, it can be stated that the main indications for PMFMRI are: termination of pregnancies in case of fetal malformations, late miscarriages, and intrauterine fetal deaths in every case where the parents refuse autopsy. It can also be performed in the cases where the parents do accept the autopsy, as PMFMRI may orient the pathologist and potentially provide additional information compared to US [12].

As mentioned, in case of termination of pregnancy, PMFMRI can confirm the antenatal findings of obstetrical US and potentially provide supplementary information. In the cases of late miscarriages and intrauterine fetal deaths, PMFMRI may help to understand their causes.

PMFMRI can be performed even in early second trimester fetuses. In our experience, even in these low weight fetuses, PMFMRI seems to be more accurate than obstetrical ultrasound in characterizing brain or fetal body malformations. The examination offers an easy evaluation of the deceased fetus. The present exceptions are cardiac malformations [11].

## The procedure

PMFMRI should be performed as soon as possible after the termination of the pregnancy or fetal death. In the Magnetic Resonance Imaging Autopsy Study (MaRIAS), all the bodies were stored in a mortuary at 4 °C for 1 to 6 days [13]. It should be kept in mind that there may be a delay between fetal death and delivery in case of termination of pregnancy. This delay could be longer after spontaneous intrauterine fetal death.

PMFMRI can be performed either at 1.5 T or 3 T. The rate of diagnostic errors is lower using 3 T than 1.5 T MR magnets, especially for low body weight fetuses and for all body parts except for the brain and orbits [14].

The examination is usually performed in the absence of the parents. In any case, detailed explanation on the examination is usually provided and, in some centers/countries, they may be asked to sign an informed consent [15]. The body should be covered and placed supine, lying as close as possible to the anatomical neutral position. The coil that will be used needs to be adapted to the size of the body. In general, a head coil is used for the brain and spine and a body coil for the body imaging [16]. Yet, in smaller fetuses, the head coil may provide enough coverage for both the head and the body. We prefer to use separate head and neck coils.

**Fig. 1:**
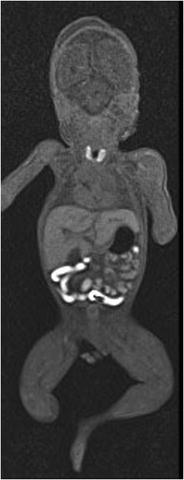
Coronal T1-weighted (W) image of a 24 weeks gestation fetus showing the normal T1 hypersignal of the meconium and thyroid. The fetal liver appears relatively hypersignal, probably due to high glycogen content

Various protocols for the examination have been proposed in the literature; we use a simplified protocol based on the one reported by Norman et al. [16]. Our protocol includes a 3D T2-weighted (W) volumetric acquisition [3D constructive interference in steady state (CISS)], of the head and whole body with reconstructions in the three planes, T1-W sequences in the sagittal and coronal planes on the head and the rest of the body, and axial T2* of the head (Table 1). Depending on the gestational age, acquisition of the head and the body can be made at the same time or separately. T1-W images have poor contrast and low signal in postmortem imaging [16]. There is a normal high T1-W signal in the normal thyroid and in the bowel (distal small then large bowel, depending on the gestational age) (Fig. 1). High signal in the bowel is related to its meconium content (Fig. 1). High-resolution T2-W images offer much better contrast for PMFMRI [3, 17]. Diffusion-weighted sequences of the fetal brain has been used to estimate the degree of maceration whenever the exact time of death is unknown. Still, it has a poor contribution in clinical routine [18].

**Table 1:** Sequence parameters for postmortem fetal magnetic resonance imaging (PMFMRI)

	3D CISS T2 (sag)	T1 TSE (sag)	T1 TSE (cor)	T2* (axial brain)
Voxel (mm)	0.8 × 0.8 × 0.8	1.4 × 0.8 × 2.5	1.4 × 0.8 × 2.5	1.1 × 0.9 × 3
Slices	96	40	40	30
Slice thickness (mm)	0.8	2.5	2.5	3
Distance factor (%)	20	25	25	15
FOV read (mm)	210	380	380	230
FOV phase (%)	68.8	75	100	100
TR (ms)	6.45	427	427	1030
TE (ms)	2.69	11	11	26
Averages	1	2	2	1
Duration of acquisition (min)	3.19	3.15	2.33	2.5
Phase oversampling (%)	13	40	100	0

FOV: field of view; CISS: constructive interference in steady state; TSE: turbo spin echo; TR: repetition time; TE: echo time; sag/cor: sagittal or coronal acquisition

The total duration of PMFMRI in our institution is around 30 min.

## Interpretation of PMFMRI: diagnosing anomalies and physiological changes

Knowledge of the antenatal sonographic findings as well as all relevant clinical or genetic data is essential before interpreting PMFMRI, as it will increase the diagnostic accuracy.

Evaluating the fetus should be standardized and systematic, analyzing all structures and organs from the head to toe. The various findings should be correlated with fetal age. Any abnormality should be analyzed and characterized as it would be on (living) fetal MRI (for second trimester fetuses) or with postnatal findings in case of third trimester fetal deaths.

**Fig. 2:**
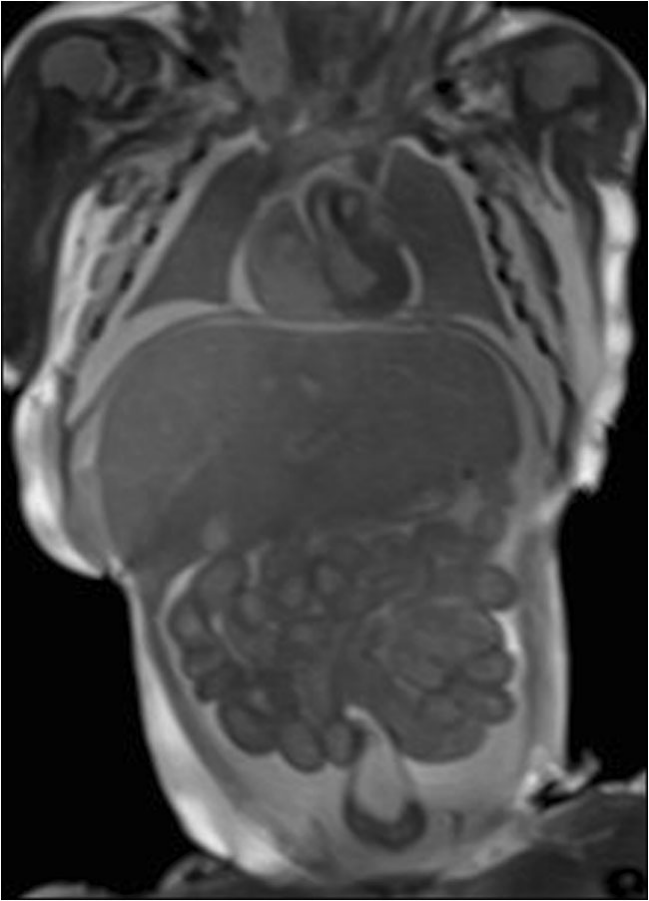
Coronal T2-W image of a 25 weeks fetus in a case of intrauterine death. There is bilateral pleural effusion, subcutaneous edema, ascitis, distended bowel loops, and enlarged appearance of the normal fetal liver. All findings are physiological postmortem changes

**Fig. 3:**
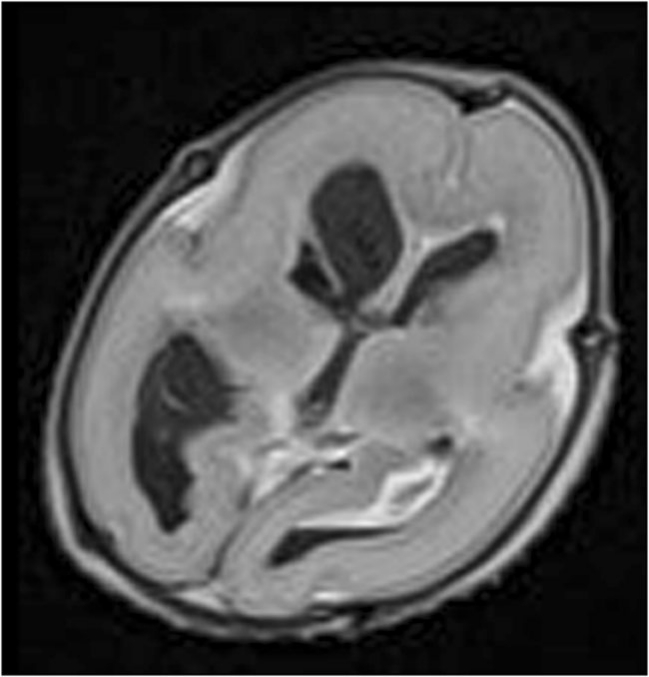
Axial T2-W image of the brain of a 20 weeks fetus showing bilateral intraventricular hemorrhage as a normal postmortem change

**Fig. 4:**
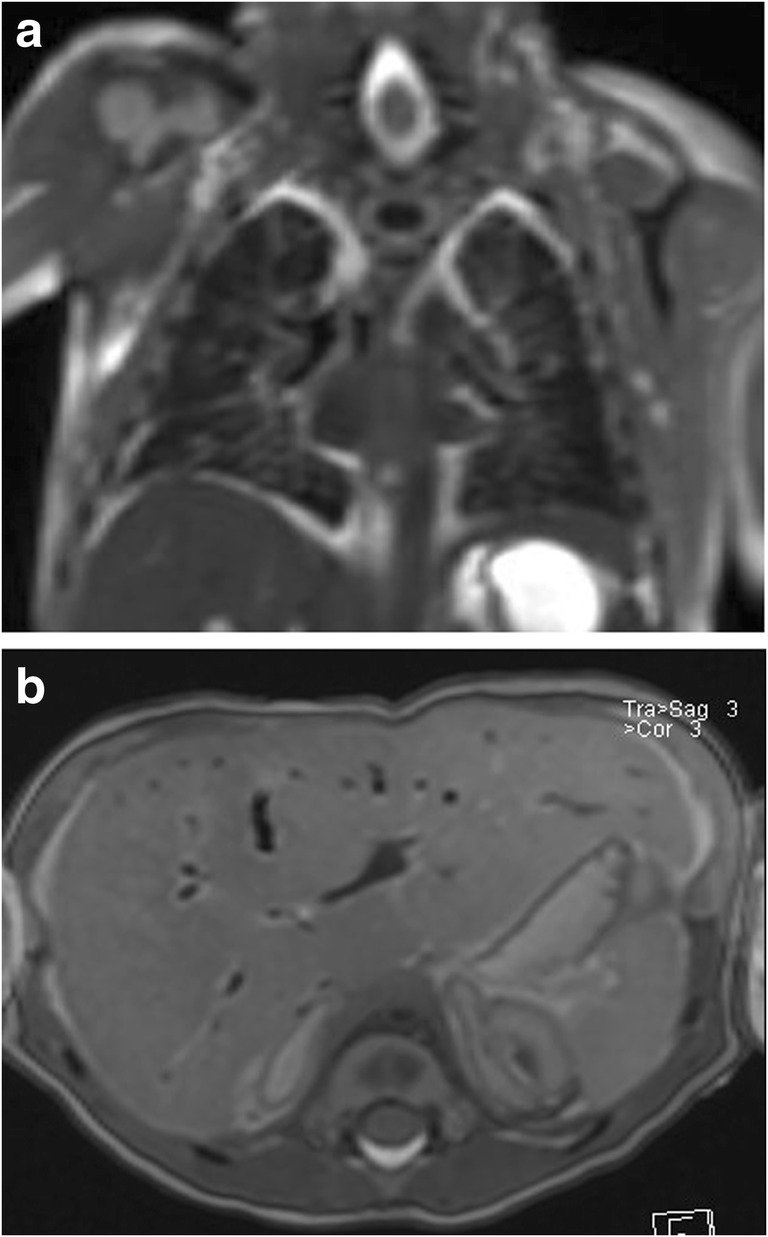
Postmortem fetal magnetic resonance imaging (PMFMRI) in a term stillbirth. Physiological postmortem changes. **a** Coronal T2-W image showing dark appearance of the lungs containing air. **b** Axial T2-W image showing air in the hepatobiliary system

**Fig. 5:**
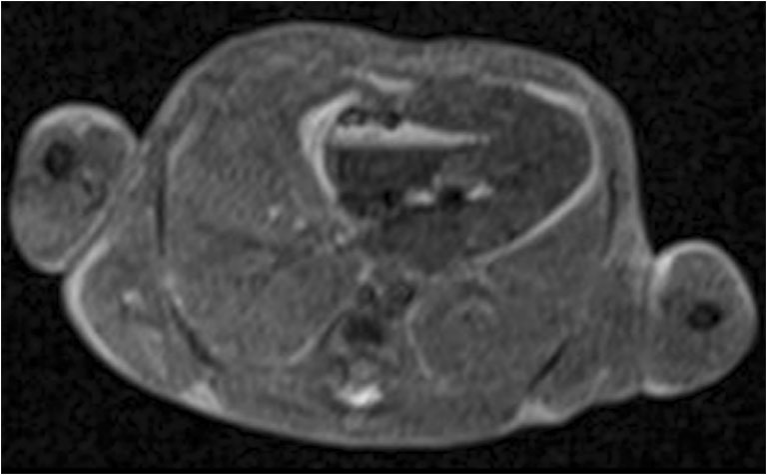
Axial T2-W image of a 23 weeks fetus showing air and blood clots in the heart as a physiological postmortem change. It also shows the normal gray appearance of fetal lungs that have not been aerated

Importantly, there are several PMFMRI changes that develop progressively after death (mainly because of the tissular lysis and maceration) that should not be misinterpreted as pathological findings [19]. The following should be mentioned, among others:

- *Fluid accumulation*: Subcutaneous edema, pleural, pericardial effusions and ascites (Fig. 2). There is often some degree of body deformation.
- *Head*: Skull deformity (that needs to be differentiated from head molding that occurs at delivery), collapsed eyeballs, brain ischemia (edema, loss of gray-white matter differentiation, and low T2 signal in basal ganglia), frontal irregular cortical plate indentation [17], tonsillar descent, venous stasis, and small intraventricular hemorrhage without dilatation (Fig. 3).
- *Chest*: Dark (hyposignal) appearances of the lungs indicate the presence of air within the airway (when the infant has breathed before death or when cardiopulmonary resuscitation methods have been used in stillbirths), whereas the lungs display an intermediate in case of termination of pregnancy or late miscarriage [20] (Fig. 4a).
- *Heart*: Small pericardial effusions, intracardiac air, blood clots, and fluid–fluid level in the heart and major vessels are common findings (Fig. 5). The cardiac ventricles may have a pseudo-thickened appearance after death; this should not be mistaken with ventricular hypertrophy [21]. Valves leaflets can be seen clearly when closed; they are more difficult to visualize if fully open.
- *Abdomen*: Changes include gas in the hepatobiliary system (Fig. 4b), small and large bowel dilatation (Fig. 2), and a pseudohepatomegaly (Fig. 2) [19].

It should be noted that the postmortem interval cannot be determined by PMFMRI even if some research has been done on the influence of various factors, such as the accumulation of fluid in the lungs or the change in brain signal [22].

## Diagnostic accuracy of PMFMRI

Since the late 1990s, when Brookes et al. published the first study on non-invasive perinatal necropsy, many advances have been achieved in PMFMRI. The largest recent prospective comparison of standard autopsy versus less invasive autopsy (postmortem MRI and ancillary investigations such as examination of the placenta and postmortem blood samplings but no incision) in fetuses and children is the so-called the Magnetic Resonance Imaging Autopsy Study (MaRIAS, Lancet 2013) [9]. In this study, the authors have analyzed 400 patients, of which 277 were fetuses. The cause of death or major pathological lesions detected by minimally invasive autopsy were concordant with conventional autopsy in 357 cases (89.3%). The concordance was even higher in fetuses: 94.6% in fetuses of less than 24 weeks gestation and 95.7% in fetuses of more than 24 weeks gestation (compared with 76.4% in children) [9].

**Fig. 6:**
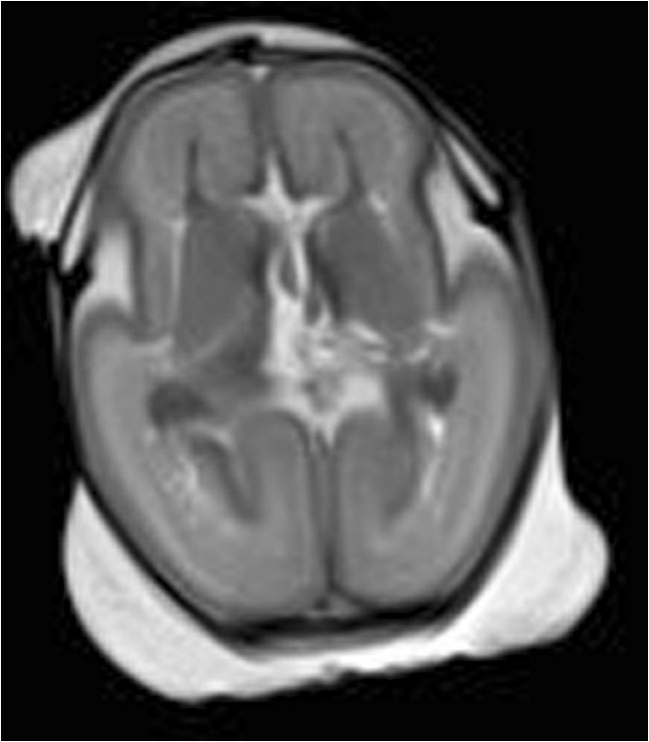
PMFMRI of a 21 weeks gestation fetus who died in utero. Axial T2-W image shows indirect signs of corpus callosum agenesis. Note that there is a subcutaneous edema that has to be considered as a normal postmortem change

**Fig. 7:**
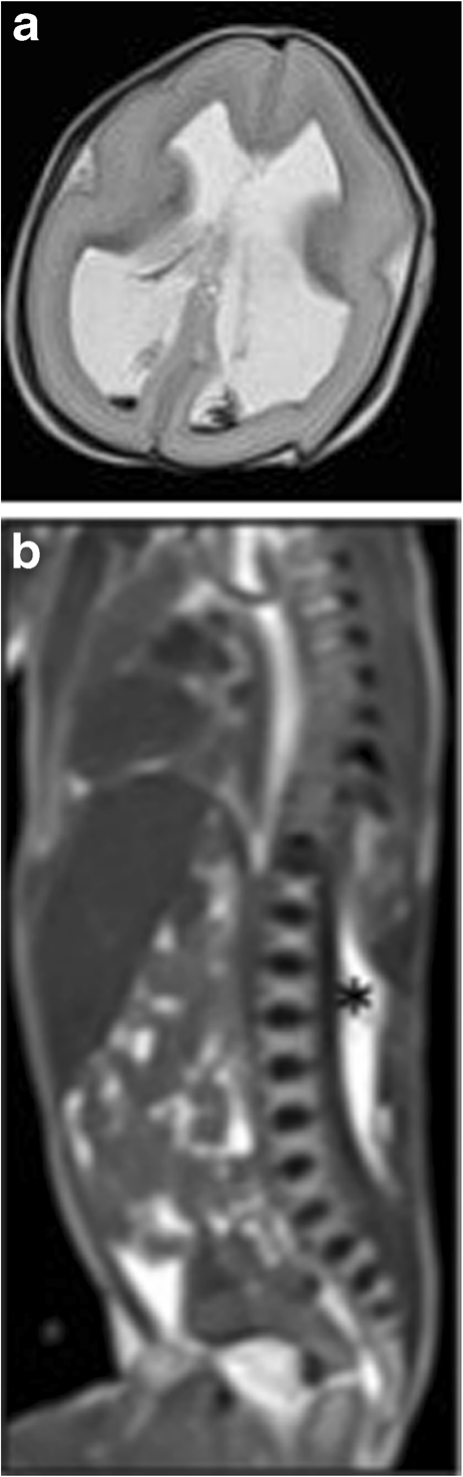
PMFMRI of a 22 weeks gestation fetus, for which the pregnancy was interrupted for extensive spinal dysraphism. **a** Axial T2-W image shows bilateral ventriculomegaly. There is a small hemorrhage in the occipital horns that is considered as a postmortem change. **b** Sagittal T2-W image shows a close spinal dysraphism (asterisk)

**Fig. 8:**
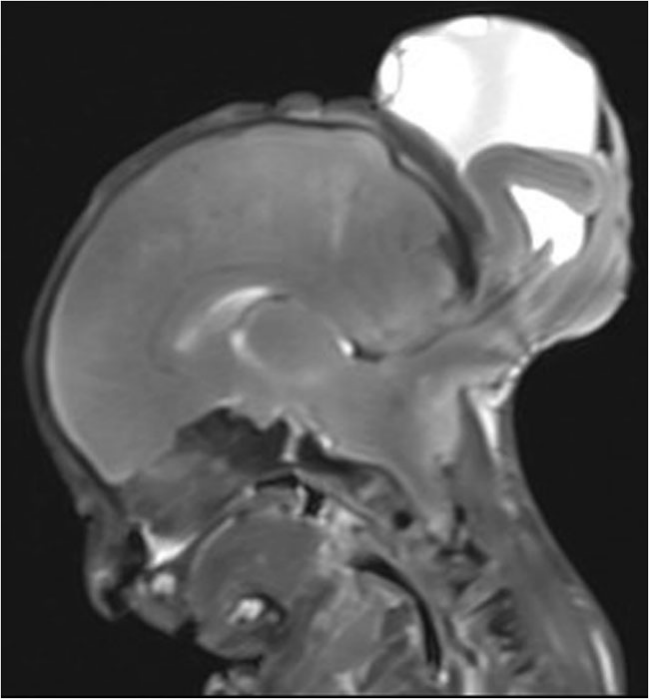
PMFMRI of a 25 weeks gestation fetus in a case of late miscarriage. Sagittal T2-W image shows an occipital encephalocele

To be noted, this study and others before have demonstrated that the diagnostic accuracy of PMFMRI varies according to the different body parts:

- *Neurological abnormalities*: PMFMRI is highly accurate for the detection of cerebral malformations (sensitivity 88.4%, specificity 95.2%) (Figs. 6, 7, and 8) and intracranial bleedings (sensitivity 100%, specificity 99.1%), but less sensitive for detecting ischemic injuries (sensitivity 68%, specificity 96.1%) [23]. It seems challenging to differentiate between premortem ischemic injuries and physiological postmortem changes.

Furthermore, PMFMRI provides important diagnostic information in 50% of fetuses in which conventional brain autopsy is non-diagnostic due to maceration and autolysis of the brain tissues [22].

**Fig. 9:**
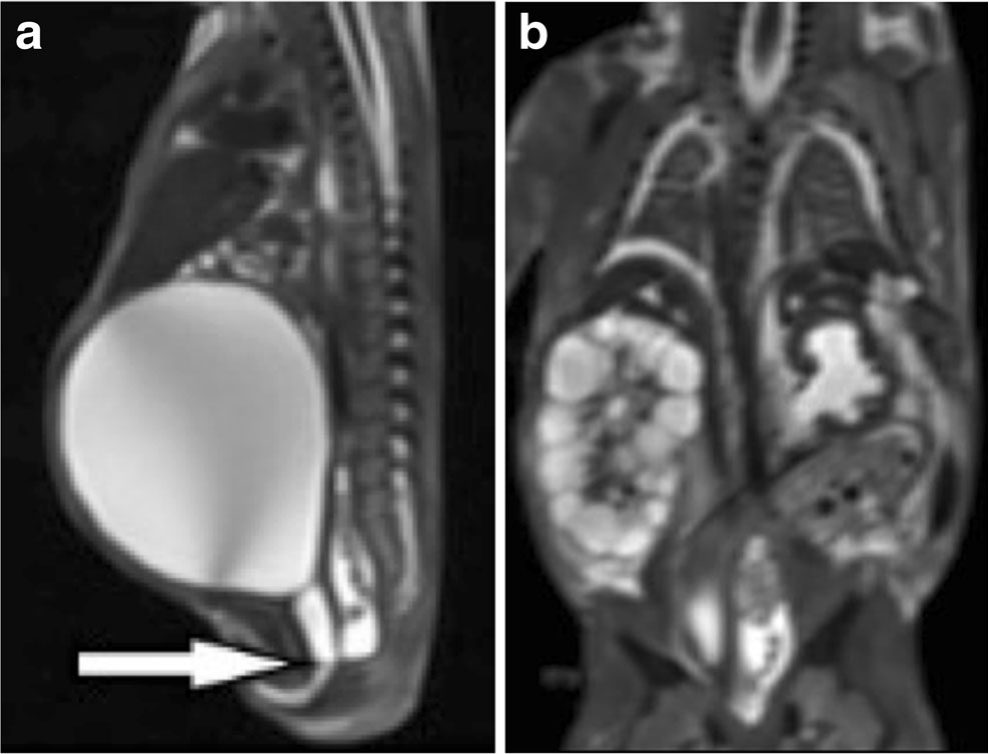
PMFMRI of an 18 weeks gestation fetus in a case of termination of pregnancy. **a** Sagittal T2-W image shows a megabladder and dilated posterior urethra above the posterior urethral valves (arrow). **b** Coronal T2-W image shows a right multicystic dysplastic kidney and left hydronephrosis

**Fig. 10:**
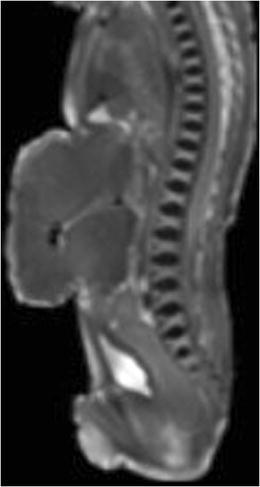
Sagittal T2-W image of a 25 weeks gestation fetus with an abdominal wall defect and a large hepatocele

**Fig. 11:**
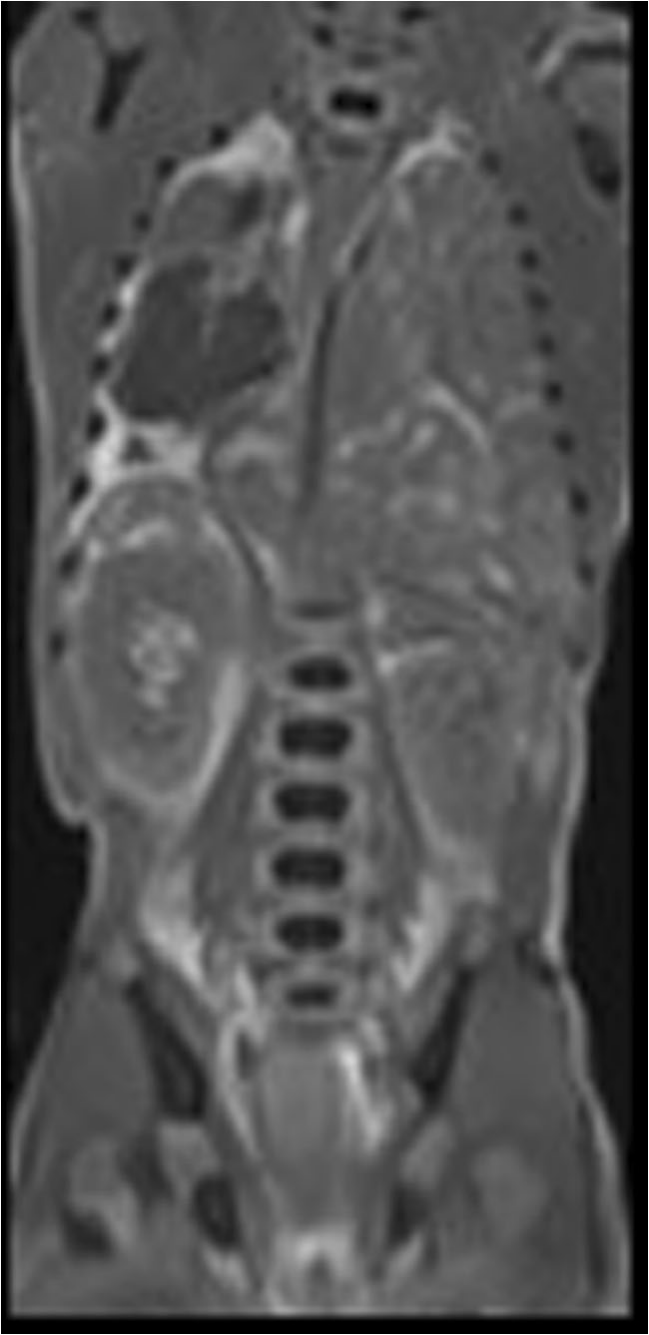
Coronal T2-W image of a 25 weeks gestation fetus with a left congenial diaphragmatic hernia

- *Abdominal abnormalities*: PMFMRI is highly accurate for the detection of renal and urinary tract abnormalities (sensitivity 80%, overall concordance 97%) and for anomalies of the abdominal wall (Figs. 9 and 10). It is less accurate for adrenal, liver, and intestinal abnormalities (sensitivity 50%) [24]. For instance, the adrenals may appear hemorrhagic-like on PMFMRI but normal on autopsy. Conversely, they may appear normal on PMFMRI but with microscopic hemorrhage on autopsy. Intestinal anomalies, such as atresia, obstruction, and malrotation, can be difficult to diagnose, since bowel dilatation can be due to a postmortem change.
- *Non-cardiac thoracic abnormalities*: In the fetus, because there is no lung aeration, a normal thoracic PMFMRI predicts normal autopsy in over 80% of the cases [21]. The overall sensitivity and specificity of PMFMRI for non-cardiac thoracic pathology is better in fetuses than in children, with, respectively, 80% (39.5% in children) and 85.5% [21]. Its sensitivity in detecting anatomical abnormalities (pleural effusion, lung or chest hypoplasia, congenital diaphragmatic hernia) is good (Fig. 11), but it is poorer at detecting infection and diffuse alveolar hemorrhage [21].
- *Cardiovascular abnormalities*: The overall sensitivity and specificity of PMFMRI are 72.7 and 96.2% for detecting any cardiac pathology [25]. The technique is able to detect structural cardiac anomalies in fetuses older than 24 weeks with a good negative predictive value [25].
- *Musculoskeletal abnormalities*: PMFMRI has a high diagnostic accuracy for the exclusion of musculoskeletal abnormalities (negative predictive value 93.8%) but its sensitivity is relatively poor (51.1%) [26]. Fetal conventional radiographs and/or fetal skeletal CT are clearly useful whenever skeletal anomalies are suspected.

Interestingly, in our experience, PMFMRI is more accurate than obstetrical ultrasound in detecting major CNS and fetal body malformations, especially during the second trimester [11].

## Advantages of PMFMRI

PMFMRI has numerous advantages:

- It provides immediate diagnosis in comparison with autopsy, whose results can take a longer time. According to the survey of the French society of perinatal pathology in 2012 (http://www.chu-clermontferrand.fr/internet/sites/soffoet/default.aspx), the delay in providing a complete histologic report is 1 month in 30% and more than 2 months in 60% of the fetuses. Noteworthy, 40% of the French pathologists in practice today will retire in less than 10 years and 80% of them will not be replaced.
- It represents an alternative to autopsy, with at least some information provided in cases where the parents do not agree to an invasive procedure.
- It can guide the autopsy in order to have less invasive procedures. If a histological assessment of tissue is required to confirm a suspected diagnosis, PMFMRI can be used to target samplings (transcutaneous needle biopsies). PMFMRI may also be combined with laparoscopic examination (keyhole techniques) to facilitate direct organ examination while minimizing incisions [3]. Both techniques (percutaneous or endoscopic tissue sampling) minimize the body disfiguration and can be more acceptable for the parents.
- Images can be stored, easily sent, and used in multidisciplinary meetings.
- Precise measurements and organs volumetries can be obtained [27].

## Limitations of PMFMRI

There are some limitations to the use of PMFMRI:

- The general accessibility of MRI is a clear limitation.
- Radiographers trained to this type of examination are not always available. Some are reluctant to perform examinations on dead fetuses.
- There is also a need for trained (pediatric) radiologists for the interpretation of PMFMRI. A good knowledge of the fetal anatomy and congenital and acquired anomalies occurring during fetal life is mandatory. Furthermore, awareness of all physiological postmortem changes is required to correctly interpret PMFMRI findings [3]. There is a learning curve for a radiologist before optimizing a report of PMFMRI. Furthermore, a recent study suggests that training on a large dataset of postmortem examinations allows a single reporter to reach a higher diagnostic accuracy [28].
- Relatively higher rate of non-diagnostic imaging examinations in early gestation fetuses.

## Future developments in postmortem imaging

Establishing guidelines and standardizing the examinations will help the diffusion of the technique. International multicentric collaboration would be helpful as well.

Research using high-field (9.4 T) MRI for PMFMRI seems very promising and may lead to increasing the accuracy of postmortem imaging [29]. This research would potentially allow better investigation of low body weight fetuses.

## Conclusion

Advances in imaging technology along with the reduction in parental acceptance of conventional autopsy are likely to change the way fetal death will be investigated. Postmortem fetal magnetic resonance imaging (PMFMRI) is likely to develop and become an important part of the fetal imaging. The use of fetal postmortem examination modalities should be decided between the different specialists involved after reviewing the full clinical history, prenatal ultrasound findings, and external examination. PMFMRI should be performed in all cases of parental autopsy refusal or prior to any histopathology examination to assess if a full autopsy or, rather, a targeted biopsy is needed.

## References

[1] Dickinson JE, Prime DK, Charles AK (2007). The role of autopsy following pregnancy termination for fetal abnormality. Aust N Z J Obstet Gynaecol.

[2] Piercecchi-Marti MD, Liprandi A, Sigaudy S (2004). Value of fetal autopsy after medical termination of pregnancy. Forensic Sci Int.

[3] Addison S, Arthurs OJ, Thayyil S (2014). Post-mortem MRI as an alternative to non-forensic autopsy in foetuses and children: from research into clinical practice. Br J Radiol.

[4] Shojania KG, Burton EC (2008). The vanishing nonforensic autopsy. N Engl J Med.

[5] Arthurs OJ, Taylor AM, Sebire NJ (2015). Indications, advantages and limitations of perinatal postmortem imaging in clinical practice. Pediatr Radiol.

[6] Ben-Sasi K, Chitty LS, Franck LS (2013). Acceptability of a minimally invasive perinatal/paediatric autopsy: healthcare professionals' views and implications for practice. Prenat Diagn.

[7] Lewis C, Hill M, Arthurs OJ, Hutchinson C, Chitty LS, Sebire NJ (2018). Factors affecting uptake of postmortem examination in the prenatal, perinatal and paediatric setting. BJOG.

[8] Arthurs OJ, van Rijn RR, Sebire NJ (2014). Current status of paediatric post-mortem imaging: an ESPR questionnaire-based survey. Pediatr Radiol.

[9] Thayyil S, Sebire NJ, Chitty LS (2013). Taylor AM; MARIAS collaborative group. Post-mortem MRI versus conventional autopsy in fetuses and children: a prospective validation study. Lancet.

[10] Jawad N, Sebire NJ, Wade A, Taylor AM, Chitty LS, Arthurs OJ (2016). Body weight lower limits of fetal postmortem MRI at 1.5 T. Ultrasound Obstet Gynecol.

[11] D’Hondt A, D’Haene N, Rommens J, Cassart M, Avni EF (2017). The contribution of mid-trimester virtual autopsy with MR imaging. Pediatr Radiol.

[12] Sarda-Quarello L, Tuchtan L, Bartoli C (2015). Post-mortem perinatal imaging: state of the art and perspectives, with an emphasis on ultrasound. Gynecol Obstet Fertil.

[13] Thayyil S, Sebire NJ, Chitty LS (2011). Post mortem magnetic resonance imaging in the fetus, infant and child: a comparative study with conventional autopsy (MaRIAS protocol). BMC Pediatr.

[14] Kang X, Cannie MM, Arthurs OJ (2017). Post-mortem whole-body magnetic resonance imaging of human fetuses: a comparison of 3-T vs. 1.5-T MR imaging with classical autopsy. Eur Radiol.

[15] Judge-Kronis L, Hutchinson JC, Sebire NJ, Arthurs OJ (2016). Consent for paediatric and perinatal postmortem investigations: implications of less invasive autopsy. J Forensic Radiol Imaging.

[16] Norman W, Jawad N, Jones R, Taylor AM, Arthurs OJ (2016). Perinatal and paediatric post-mortem magnetic resonance imaging (PMMR): sequences and technique. Br J Radiol.

[17] Scola E, Conte G, Palumbo G (2018). High resolution post-mortem MRI of non-fixed in situ foetal brain in the second trimester of gestation: normal foetal brain development. Eur Radiol.

[18] Papadopoulou I, Langan D, Sebire NJ, Jacques TS, Arthurs OJ (2016). Diffusion-weighted post-mortem magnetic resonance imaging of the human fetal brain in situ. Eur J Radiol.

[19] Arthurs OJ, Barber JL, Taylor AM, Sebire NJ (2015). Normal perinatal and paediatric postmortem magnetic resonance imaging appearances. Pediatr Radiol.

[20] Barber JL, Sebire NJ, Chitty LS, Taylor AM, Arthurs OJ (2015). Lung aeration on post-mortem magnetic resonance imaging is a useful marker of live birth versus stillbirth. Int J Legal Med.

[21] Arthurs OJ, Thayyil S, Olsen OE (2014). Owens CM; magnetic resonance imaging autopsy study (MaRIAS) collaborative group. Diagnostic accuracy of post-mortem MRI for thoracic abnormalities in fetuses and children. Eur Radiol.

[22] Arthurs OJ, Hutchinson JC, Sebire NJ (2017). Current issues in postmortem imaging of perinatal and forensic childhood deaths. Forensic Sci Med Pathol.

[23] Arthurs OJ, Thayyil S, Pauliah SS (2015). Diagnostic accuracy and limitations of post-mortem MRI for neurological abnormalities in fetuses and children. Clin Radiol.

[24] Arthurs OJ, Thayyil S, Owens CM (2015). Magnetic resonance imaging autopsy study (MaRIAS) collaborative group. Diagnostic accuracy of post mortem MRI for abdominal abnormalities in foetuses and children. Eur J Radiol.

[25] Taylor AM, Sebire NJ, Ashworth MT (2014). Postmortem cardiovascular magnetic resonance imaging in fetuses and children: a masked comparison study with conventional autopsy. Circulation.

[26] Arthurs OJ, Thayyil S, Addison S (2014). Diagnostic accuracy of postmortem MRI for musculoskeletal abnormalities in fetuses and children. Prenat Diagn.

[27] Breeze AC, Gallagher FA, Lomas DJ, Smith GC, Lees CC, Cambridge Post-Mortem MRI Study Group (2008). Postmortem fetal organ volumetry using magnetic resonance imaging and comparison to organ weights at conventional autopsy. Ultrasound Obstet Gynecol.

[28] Ashwin C, Hutchinson JC, Kang X (2017). Learning effect on perinatal post-mortem magnetic resonance imaging reporting: single reporter diagnostic accuracy of 200 cases. Prenat Diagn.

[29] Hutchinson JC, Arthurs OJ, Sebire NJ (2016). Postmortem research: innovations and future directions for the perinatal and paediatric autopsy. Arch Dis Child Educ Pract Ed.

